# Analysis of the levels of lysine-specific demethylase 1 (*LSD1*) mRNA in human ovarian tumors and the effects of chemical LSD1 inhibitors in ovarian cancer cell lines

**DOI:** 10.1186/1757-2215-6-75

**Published:** 2013-10-29

**Authors:** Sergiy Konovalov, Ivan Garcia-Bassets

**Affiliations:** 1 Department of Medicine, School of Medicine, University of California, San Diego, La Jolla, CA, USA

**Keywords:** Human ovarian tumors, LSD1/KDM1A/AOF2, RT-qPCR, TCGA, Transcriptomic signature, Cell cycle, Immune response, Inflammatory response, Subtype C5, LSD1 inhibitors

## Abstract

**Background:**

Lysine-specific demethylase 1 (LSD1, also known as KDM1A and AOF2) is a chromatin-modifying activity that catalyzes the removal of methyl groups from lysine residues in histone and non-histone proteins, regulating gene transcription. LSD1 is overexpressed in several cancer types, and chemical inhibition of the LSD1 activity has been proposed as a candidate cancer therapy. Here, we examine the levels of *LSD1* mRNA in human ovarian tumors and the cytotoxicity of several chemical LSD1 inhibitors in a panel of ovarian cancer cell lines.

**Methods:**

We measured *LSD1* mRNA levels in a cohort of n = 177 normal and heterogeneous tumor specimens by quantitative real time-PCR (qRT-PCR). Tumors were classified by FIGO stage, FIGO grade, and histological subtypes. We tested the robustness of our analyses in an independent cohort of n = 573 serous tumor specimens (source: TCGA, based on microarray). Statistical analyses were based on Kruskal-Wallis/Dunn’s and Mann Whitney tests. Changes in *LSD1* mRNA levels were also correlated with transcriptomic alterations at genome-wide scale. Effects on cell viability (MTS/PMS assay) of six LSD1 inhibitors (pargyline, TCP, RN-1, S2101, CAS 927019-63-4, and CBB1007) were also evaluated in a panel of ovarian cancer cell lines (SKOV3, OVCAR3, A2780 and cisplatin-resistant A2780cis).

**Results:**

We found moderate but consistent *LSD1* mRNA overexpression in stage IIIC and high-grade ovarian tumors. *LSD1* mRNA overexpression correlated with a transcriptomic signature of up-regulated genes involved in cell cycle and down-regulated genes involved in the immune/inflammatory response, a signature previously observed in aggressive tumors. In fact, some ovarian tumors showing high levels of *LSD1* mRNA are associated with poor patient survival. Chemical LSD1 inhibition induced cytotoxicity in ovarian cancer lines, which roughly correlated with their reported LSD1 inhibitory potential (RN-1,S2101 >> pargyline,TCP).

**Conclusions:**

Our findings may suggest a role of LSD1 in the biology of some ovarian tumors. It is of special interest to find a correlation of *LSD1* mRNA overexpression with a transcriptomic signature relevant to cancer. Our findings, therefore, prompt further investigation of the role of LSD1 in ovarian cancer, as well as the study of its enzymatic inhibition in animal models for potential therapeutic purposes in the context of this disease.

## Background

Lysine-specific demethylase 1 (LSD1, also known as KDM1A, AOF2, BHC110, and KIA0601) is a nuclear enzymatic activity that catalyzes the removal of methyl groups from histones and non-histone lysine residues [1-5]. LSD1 demethylates mono- and di-methylated histone H3 at lysines 4 and 9 (H3K4me1/2 and H3K9me1/2, respectively) mediating the transcriptional actions of hormone-liganded nuclear receptors [6-9], oncogene c-myc [10], and Snail1 [11-13], among others. LSD1 also demethylates non-histone substrates, such as tumor suppressor p53 and cell cycle and apoptosis regulator E2F1 [14,15]. LSD1 has been found overexpressed in liver cancer [16], gastric cancer [17], breast cancer [18,19], bladder, lung and colorectal cancers [20], Ewing's sarcoma [21], and neuroblastoma [22]; and overexpression of LSD1 is a predictor of poor prognosis in prostate and liver cancer [16,23]. Together, these and other studies suggest that LSD1 is linked to cancer and could be a target for drug discovery [24,25].

LSD1 is a flavin adenine dinucleotides-dependent (FAD) amine oxidase [1]. Thus, FDA-approved inhibitors of FAD amine oxidases such as mitochondrial-associated monoamine oxidase (MAO) A and B and polyamine oxidase (PAO) are non-selective inhibitors of the LSD1 activity [4]. Recently, more potent and selective LSD1 inhibitors have been developed, which can be grouped into four different classes based on their chemical structure. One class is based on analogues of (bis)guanidine and (bis)biguanide polyamines and oligoamines [26]. It includes compound CAS 927019-63-4, which is a polyamine analog that selectively inhibits LSD1 *in vitro* and that induces re-expression of aberrantly silenced genes in human colon carcinoma cells [27,28]. Polyamine analogs have also been tested in breast cancer cells [29]. A second class of LSD1 inhibitors is based on tranylcypromine (TCP, also known as 2-phenylcyclopropylamine or 2-PCPA). TCP is a non-selective and irreversible MAO inhibitor that forms a covalent adduct with FAD. FAD-approved TCP has been used in the clinical treatment of mood and anxiety disorders [30-32]. Two of the most recently developed TCP-analogues with LSD1 inhibitory activity are S2101 [33] and RN-1 [34]. A third class of LSD1 inhibitors is based on FDA-approved antidepressants pargyline and phenelzine [25]. The LSD1 inhibitory activity of pargyline has been tested in prostate cancer cells [7]. Finally, a fourth class of LSD1 inhibitors mimics the peptide structure of LSD1 substrates, such as histone H3 tails [35,36]. For example, CBB1007 is an amidino-guanidinium compound that was developed based on the crystal structure of LSD1 associated with a peptide inhibitor derived from the N-terminal tail of H3 [36]. CBB1007 is a potent and reversible substrate competitive inhibitor of LSD1 that *in vitro* arrests pluripotent cancer cells with minimal effect on non-pluripotent cancer or normal somatic cells [36].

Despite the abundant literature on LSD1 and the many studies testing LSD1 inhibition in cancer cells, the levels of LSD1 expression in human ovarian tumors and the effects of LSD1 inhibitors in ovarian cancer cells have not yet been investigated. In this study, we examine the levels of *LSD1* mRNA expression in two independent cohorts of human ovarian tumors. One analysis is based on quantitative real-time PCR (qRT-PCR) and n = 177 specimens, and the other analysis is based on microarray and n = 573 specimens (source: The Cancer Genome Atlas or TCGA). We also examine transcriptomic profiles associated with changes in levels of *LSD1* mRNA in TCGA tumors. Finally, we examine the effects on cell viability of six chemical LSD1 inhibitors (CAS 927019-63-4, TCP, RN-1, S2101, pargyline, and CBB1007) in a panel of ovarian cancer cell lines. Our study is the first systematic analysis of LSD1 in the context of ovarian cancer.

## Methods

### Human cohort of ovarian normal and tumor samples

Ovarian Cancer cDNA Tissue Scan™ was purchased from OriGene Technologies (Rockville, MD, USA). It contains n = 192 ovarian normal and tumor cDNA samples (plate HORT101, Lot#1210; plate HORT102, Lot#0712; plate HORT103, #Lot 0210; and plate HORT104, Lot#0210). For our studies, we reduced the number of specimens in the cohort to n = 177 (see section below, ‘qRT-PCR-based measurement of *LSD1* mRNA expression in our study cohort*’* for details on how we filtered these data). The clinicopathologic parameters and histological and clinical information of the final cohort can be found at the OriGene website. A summary of this information can also be found in Additional file 1: Table S1.

### Cell lines

Human ovarian cancer cell lines SKOV3 and OVCAR3 were purchased from the American Type Culture Collection (ATCC, Manassas, VA, USA). Human ovarian cancer cell lines A2780 and its cisplatin-resistant clone (A2780-cis) were purchased from Sigma-Aldrich (St. Louis, MO, USA). Human breast cancer MCF7 and prostate cancer LNCaP cells were generously provided by Dr. Michael G. Rosenfeld (University of California, San Diego, CA, USA). SKOV3 cells were cultured in McCoy’s 5A Modified Medium (16600–108; Life Technologies, Carlsbad, CA, USA) containing 10% fetal bovine serum, or FBS (Omega Scientific, Tarzana, CA, USA). OVCAR3 cells were cultured in RPMI-1640 Modified Medium (30–2001; ATCC) containing 20% FBS supplemented with 10 μg/mL bovine insulin (Sigma-Aldrich). A2780 and A2780-cis cells were cultured in RMPI-1640 + GlutaMAX-I medium (61870–127; Life Technologies) containing 10% FBS and supplemented with 25 mM HEPES buffer. MCF7 cells were cultured in DMEM(1x) + GlutaMAX-I medium (10566–024; Life Technologies) containing 10% FBS. LNCaP cells were cultured in Advanced DMEM/F12(1x) medium (12634–028; Life Technologies) containing 10% FBS. Trypsin/EDTA (Life Technologies) was used for detachment of SKOV3, MCF7, and LNCaP cells. Accutase (Innovative Cell Technologies, San Diego, CA, USA) was used for the detachment of OVCAR3, A2780, and A2780cis cells. Cells did not exceed 20 passages in any of the experiments shown in this study. Cells were maintained in cell incubators at 37°C and 5% CO_2_.

### Chemotherapeutics

LSD1 inhibitors pargyline hydrochloride (also known as pargyline) and trans-2-phenylcyclopropyl-amine hydrochloride (also known as TCP or 2-PCPA) were purchased from Sigma-Aldrich. RN-1 hydrochloride (also known as RN-1 or LSD1 Inhibitor IV, cat. # 489479), S2101 (also known as LSD1 Inhibitor II, cat. # 489477), CBB1007 (also known as LSD1 inhibitor III, cat. # 489478), and CAS 927019-63-4 (also known as LSD1 inhibitor, cat. # 489476) were purchased from Calbiochem (EMD-Millipore, Billerica, MA, USA). Chemicals were dissolved in water at a final concentration of 100 mM for pargyline and TCP, 6 mM for RN-1, and 10 mM for CAS 927019-63-4; or were dissolved in dimethyl sulfoxide (DMSO) at a final concentration of 100 mM for S2010 and 10 mM for CBB1007. Cisplatin was purchased from Sigma-Aldrich and dissolved in dimethylformamide at a final concentration of 40 mM. Dilutions were prepared in the same solvents. Solutions were made fresh in every experiment for pargyline, TCP, and cisplatin. For the rest of chemicals, stock solutions were prepared and stored at -20°C, being thawed/frozen no more than 3 times.

### qRT-PCR-based measurement of *LSD1* mRNA expression in our study cohort

Dry cDNA pellets from ovarian normal and tumor tissues (see section above, ‘Human cohort of ovarian normal and tumor samples’) were dissolved in 31 μL water at 50°C for 15 minutes, vortexing every 5 minutes. After short spinning, two aliquots of 13 μL and two aliquots of 2 μL were taken for analysis of human *LSD1* and *ACTB* mRNA expression, respectively. The four aliquots were brought to a final volume of 30 μL with 2xFastStart Universal SYBR Green Master mix (Roche Applied Science, Indianapolis, IN, USA) and the solution with primers. Primer sequences were: *LSD1*-sense 5’-GCTCGGGGCTCTTATTCCTA-3’ and *LSD1*-antisense 5’-CCCAAAAACTGGTCTGCAAT-3’ and *ACTB*-sense 5’-GGACTTCGAGCAAGAGATGG-3’ and *ACTB*-antisense 5’-AGCACTGTGTTGGCGTACAG-3’. The expected sizes of the *LSD1* and *ACTB* PCR amplicons were confirmed by agarose gel. The validity of the *LSD1* primers was also confirmed by the observation of a significant downregulation in the levels of a PCR amplicon obtained in cDNA samples from cancer cell lines treated with *LSD1* siRNA when compared to control siRNA treatment (data not shown). Standard quantitative real-time PCR (qRT-PCR) reactions were conducted in a 3000 MxPro Instrument (Agilent Technologies, Santa Clara, CA, USA), in 96-well format with adhesive film. PCR settings were the following: 2 min 50°C, 10 min at 95°C followed by 40 cycles of 95°C for 15 sec, 58°C for 15 sec, and 25 sec for 72°C. Cycle threshold (Ct) values were extracted with MxPro qPCR Software (Agilent Technologies) and calculated the difference (ΔCt) between replicates. Those samples in which ΔCt was higher than 0.5, either for *LSD1* or control *ACTB*, were excluded from our analysis, which ensured the inclusions of only robust expression measurements in further analyses. A total of n = 177 out of 192 samples (92.2%) reached this quality requirement.

### Analysis of TCGA data

Gene expression data on n = 573 ovarian serous cystadenocarcinoma (OV) samples were downloaded from the TCGA website (datasets UNC_AgilentG4502A_07_2 and UNC_AgilentG4502A_07_3). Matching clinical data were downloaded from the UCSC Cancer Browser [37] (datasets AgilentG4502A_07_2 and AgilentG4502A_07_3, version: 2013-06-03). Gene expression data for normal ovarian tissue (n = 47) were downloaded from the TCGA website. Of the normal samples, n = 8 belong to “Solid tissue normals” from the “Ovarian Cancer” dataset, and n = 39 belong to “Control” with gene expression data over a total of 546 samples available from the same dataset. The downloaded data were mined with custom Linux scripts to extract data and create tables of gene expression and clinical features for the ovarian tissue (normal and cancerous) samples. Two groups of samples were extracted according to the expression of LSD1 (*AOF2*): the first group (“*LSD1*-underexpressed”) included subjects having *LSD1* expression under 0.2 percentile, and the second group (“*LSD1*-overexpressed”) included subjects having *LSD1* expression over 0.8 percentile of all samples. Both groups included equal number of samples, n = 115. Gene expression data for the two groups were then imported into R statistical software, and SAM (Significance Analysis for Microarray)-analysis was performed on the data to determine the genes significantly differentially expressed between the groups. The significant genes fell into two groups: genes whose pattern of expression positively correlated with that of *LSD1* (i.e. they were overexpressed in the *LSD1*-overexpressed group and underexpressed in the *LSD1*-underexpressed group), and genes whose pattern of expression negatively correlated with that of LSD1 (i.e. they were underexpressed in the LSD1-overexpressed group and overexpressed in the LSD1-underexpressed group). SAM analysis (delta = 3, FDR = 3e-05) revealed n = 821 (plus *LSD1* itself) genes whose expression correlated with that of *LSD1* with the fold change >1.5 (<1/1.5 for negatively correlated genes), including n = 430 positively correlated, and n = 391 negatively correlated genes. This gene set was further filtered to only include the genes that showed certain consistency in their correlation with *LSD1* expression; namely, the genes that correlated with the direction of changes in *LSD1* expression in more than 50% of high *LSD1*-expressing samples *and* in more than 50% of low *LSD1*-expressing samples. The filtered gene set contained n = 458 genes (plus *LSD1*): n = 243 positively correlated and n = 215 negatively correlated with *LSD1* expression. For each gene, the percent of consistency (percent of samples that exhibited the consistent expression of this gene with *LSD1*) within *LSD1*-underexpressed and *LSD1*-overexpressed groups was calculated (Additional file 2: Table S2). The expression data for the filtered gene set (n = 458) was normalized to (−1:1), hierarchically clustered by centered correlation method, and then visualized as a heatmap using Cluster 3.0/Treeview software [38]. The gene expression data was then manipulated and sorted using Kingsoft Office and Apache OpenOffice software, according to clinical stages, histological subtype, gene expression, and survival data. The statistical analysis of the groups was performed in GraphPad Prism 6 for Windows (for procedures, see Statistical analysis below). Kaplan-Meier analysis was performed on three groups of samples: the *LSD1*-underexpressed (n = 115), *LSD1*-overexpressed (n = 115), and the rest of the samples in the dataset (n = 343). The statistical difference between the survival curves was calculated by Mantel-Cox survival test.

### Protein extraction and Western blot analysis

For LSD1, protein was isolated directly from culturing 10-cm plates after washing with PBS buffer and adding 400 μL modified IPH buffer (50 mM Tris–HCl, pH 8.0, 300 mM NaCl, 5 mM EDTA, 0.5% (by volume) NP-40, and inhibitor cocktail). Protein extracts were then collected after 30 min lysis at 4°C, and centrifuged for 10 min at 14.000 rpm (4°C). For PARP1 and histones, protein was isolated from 6-well plates in 250 μL modified IPH buffer (as above but 420 mM NaCl). Protein concentration was measured by the standard Bradford Protein assay. Twenty μg (for LSD1) and 7 μg (for PARP1 and H3K4me2) protein were loaded and run on 4-12% Bis-Tris gels with MES running buffer (Life Technologies). After transfer onto 0.2 μm-pore PVDF (BioRad) or nitrocellulose (Whatman) membranes, membranes were blocked with Odyssey blocking solution (LI-COR bioscience) or 5% milk/TBST for 30 min and probed with antibodies (diluted in 5% BSA/TBST) against LSD1 (17721, Abcam), tubulin (DM1A, Sigma), or H3K4me2 (39141, Active Motif), PARP1 (9542, Cell Signaling), actin (MAB1501, Chemicon), respectively, overnight at 4°C. Immunodetection was achieved after incubation with infrared (IR)-dye-conjugated (LI-COR Bioscience, Lincoln, NE, USA) or HRP-conjugated (Invitrogen, Carlsbad, CA, USA), respectively, goat anti-mouse or goat anti-rabbit diluted 1:5,000 in blocking solution. IR-dye immunoreactive bands were scanned using Odyssey Imaging System (LI-COR Bioscience) following manufacturer’s instructions. HRP signal was detected by ECL (Amersham-GE, Pittsburgh, PA, USA) and autoradiography film.

### Microscopy

Bright-field images were taken in a Nikon Diaphot microscope at a 10x magnification. Image acquisition was performed with a Canon Digital Rebel XTi camera, using ZoomBrowser EX5.7 and EOS Utility software (Canon) for remote picture shooting.

### Cell viability assay

Cells were seeded at a 1/12 dilution in 96-well plates (200 μL/well) from 10 cm confluent plates. After 24 hrs, 100 μL medium was removed and replaced with 100 μL fresh medium containing the indicated small compound concentrations in figure legends. After additional 48 hrs, 150 μL medium was removed and replaced with 30 μL medium plus 20 μL of MTP/PMS (20:1, v:v) solution We purchased MTS [3-(4,5-dimethylthiazol-2-yl)-5-(3-carboxymethoxyphenyl)-2-(4-sulfophenyl)-2H-tetrazolium, inner salt] from Promega (Madison, WI, USA), and PMS (phenazine methosulfate) from Sigma-Aldrich. Plates were maintained in cell incubators at 37°C (5% CO_2_) for 2–2.30 hours and, afterwards, absorbance was measured at 490 nm in a microQuant plate reader (Biotek Instruments, Winooski, VT, USA). Values were obtained with the KCjunior Software (Biotek). Wells in the periphery of each 96-well plate were not used for measurements (A1-A12, H1-12, B-G1, and B-G12). Each experiment was performed in triplicate with six replicates each (total data point, n = 18). We show a representative example of one of them. For estimation of IC50 values and visualization we used Excel (Microsoft) and Prism 6 (GraphPad) software. Cells under vehicle conditions were used to determine 100% viability (C1). Wells without cells (in the case of pargyline and TCP treatments) or at conditions in which no cells remained alive after treatment (visually determined, in the case of RN-1 and S2101) were used to determine 0% viability (C2). Viability percentage (C3) was calculated as: ((C3-C2)/(C1-C2))*100 in Excel. Cell viability graph show the average (mean) value of viable cells under each condition, ± s.e.m. After exporting these data to Prism, half maximum inhibitory concentration (IC50, or best fit) values and 95% confidence intervals (95% CI) were then estimated by calculating the nonlinear regression (curve fit, dose response-inhibition variable slope) with Prism 6.

### Statistical analysis

Statistical analysis in our sample cohort was conducted with the GraphPad Prism 6 version for Mac (GraphPad). For two-groups-only comparisons (n = 2), we conducted two-tailed Mann Whitney tests. For analysis of multiple groups (n > 2), we conducted one-way non-parametric ANOVA (Kruskal-Wallis test) followed by the Dunn’s *post hoc* test (unless indicated otherwise, *p*-values were calculated comparing the mean of each group to the mean of control or normal tissue). For the Kaplan-Meier analysis, we used Mantel-Cox survival test. Differences were considered significant at p≤0.05 (*). Other values were indicated as: p≤0.01 (**), p≤0.001 (***), and p≤0.0001 (****).

## Results and discussion

### qRT-PCR-based profiling of *LSD1* mRNA in human ovarian specimens

**Figure 1:**
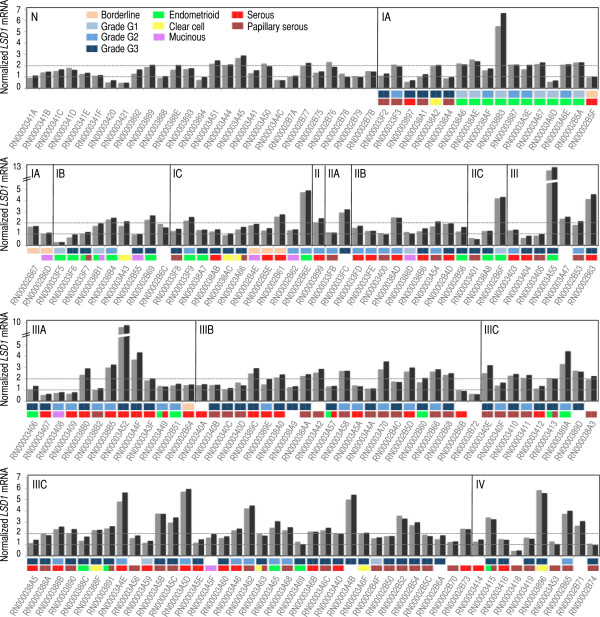
**Analysis of *LSD1 *mRNA by qRT-PCR in our study cohort reveals robus *LSD1 *mRNA levels in all ovarian normal and tumor specimens tested.** qRT-PCR analysis of *LSD1* mRNA levels in n = 177 ovarian specimens. ‘N’ indicates bulk ovarian normal tissue. ‘I-IV’ indicates FIGO stage. Two *LSD1* mRNA replicates were measured per specimen (shown in grey and black) and their values were normalized to the levels of *ACTB* mRNA measured in the same specimen also in duplicate by qRT-PCR. The y-axis refers to the relative levels of *LSD1* mRNA with respect to *ACTB* mRNA (as a percentage). The x-axis includes tumor identification numbers. FIGO grade: borderline (cantaloupe), grade G1 (light blue), grade G2 (blue), and grade G3 (dark blue); as well as tumor histological subtype: endometrioid (green), clear cell (yellow), mucinous (purple), serous (red), and papillary serous (cayenne) are indicated on top of each tumor identification number (when available). In a few cases (as indicated), specimens are classified in more than one histological subtype.

In order to accurately evaluate the levels of *LSD1* mRNA in ovarian tumors, we profiled a study cohort of n = 192 specimens by quantitative real time PCR (qRT-PCR). This cohort contained all the major ovarian cancer histological subtypes (serous, endometrioid, clear cell, and mucinous). We measured two replicates per specimen and eliminated n = 15 cases (7.8%) in which the difference between replicates exceeded a certain quality threshold (see Methods for more details). This strategy allowed us to focus our further analyses on a cohort of n = 177 specimens in which all measurements were robust. A summary of clinicopathologic features of this cohort can be found in Additional file 1: Table S1, and a list of case-by-case clinicopathologic and morphological features can be consulted on-line (see Methods for directions). *LSD1* mRNA was clearly detectable in all cases with a mean expression value of 2% relative to *ACTB* mRNA (Figure 1). The lowest expression value was 0.3% (case ID = RN000033F5, which corresponded to an endometrioid tumor classified as stage I and grade G1; second panel in Figure 1), and the highest expression value was 12.7% (case ID = RN00003A55, which also corresponded to an endometrioid tumor, but classified as stage III and grade G3; second panel in Figure 1). To our knowledge, this is the first dataset of *LSD1* mRNA measurements based on qRT-PCR obtained in a human cohort of ovarian tumors. We use this valuable dataset to study with high sensitivity the expression pattern of the *LSD1* gene in ovarian cancer.

### Moderate *LSD1* mRNA overexpression in stage IIIC and grades G2/G3 ovarian tumors

**Figure 2:**
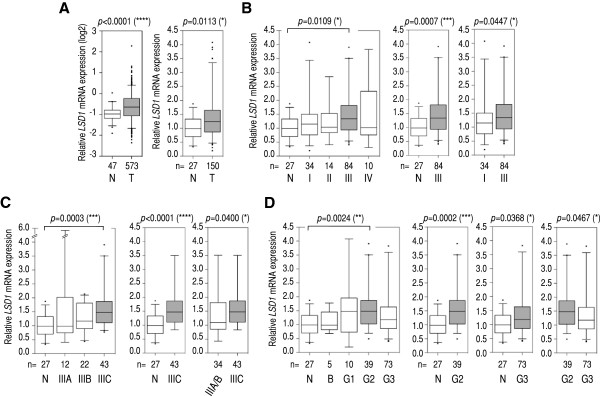
**Multi- and pair-comparison statistical tests suggest moderate *LSD1 *mRNA overexpression in stage IIIC and grade G2/G3 specimens in our cohort. (A)** Left panel: pair-comparison analysis of *LSD1* mRNA levels in normal (N) and tumor (T) specimens (serous cystadenocarcinoma, TCGA cohort; log2-scale). Right panel: as in (**A**) but in our cohort (all tumor histological subtypes; see Additional file 3: Figure S1 for a separated analysis). **(B)** Left panel: multi-comparison analysis of normal tissue (N) and tumors classified by FIGO stage (I-IV). Middle and right panels: pair-comparison analyses of normal tissue (N) and stage III tumors, and stage I and stage III tumors, respectively. **(C)** Left panel: multiple-comparison analysis of normal tissue (N) and tumors classified as stage IIIA-IIIC. Middle and right panels: pair-comparison analyses of normal tissue (N) and stage IIIC tumors; and a pool of stage IIIA and IIIB tumors (IIIA/IIIB) and stage IIIC tumors, respectively. **(D)** Left panel: multiple-comparison analysis of normal tissue (N) and tumors classified by FIGO grade: borderline **(**B**)** and G1-G3. Rest of panels: left, pair-comparison analyses of normal tissue (N) and grade G2 tumors; middle, normal tissue (N) and grade G3 tumors; and right, G2 tumors and G3 tumors. We applied the Mann–Whitney test in pair-comparison analyses, and the Kruskal-Wallis (non-parametric ANOVA) test followed by *post hoc* Dunn’s analysis in multiple-comparison analyses (number of comparisons = 10 in **B** and **D**, and 6 in **C**). *P*-values and number of specimens are shown on top and at the bottom of each panel. Two outliers exceeded the limits of the y-axis (values = 8.3 and 5.9) in some panels. In **B**-**D**, all histological subtypes were analyzed excluding mucinous. Whiskers in box plots represent 5–95 percentile values, and horizontal lines within boxes represent median values. *P*-value < 0.05 (*), *p*-value < 0.01 (**), *p*-value < 0.001 (***), and *p*-value < 0.0001 (****).

Before performing a comprehensive analysis of our study cohort, we mined The Cancer Genome Atlas (TCGA) database to obtain initial clues about the potential existence of alterations in the levels of *LSD1* mRNA in ovarian tumors (TCGA contains only ovarian tumors of the serous histological subtype). A pair-comparison analysis (Mann–Whitney) of normal tissue (n = 47) and tumors (n = 573) revealed moderate but highly significant overexpression of *LSD1* in tumor specimens (*p* < 0.0001; Figure 2A, left panel). We also divided our cohort in normal tissue (n = 27) and tumor (n = 150) specimens observing a similar result (*p* = 0.0113 by Mann–Whitney; Figure 2A, right panel). We also separated our cohort by tumor histological subtypes, observing higher levels of *LSD1* mRNA in serous, papillary serous, endometrioid, and clear cell tumors, but not in those of the mucinous subtype (*p* = 0.0296, *p* = 0.0315, *p* = 0.0421, *p* = 0.0131, and non-significant, respectively, by Mann Whitney; Additional file 3: Figure S1; first and second row of panels). Some of these differences were also observed when serous, papillary serous, endometrioid, and clear cell tumors were directly compared to mucinous specimens (Mann Whitney; Additional file 3: Figure S1; third row of panels). In the past, mucinous tumors have been reported as largely distinguishable at molecular level from the other histological subtypes [39]. Our result may further support these differences. However, we should take this observation with caution because the mucinous group consists of a small number of tumors (n = 5) in our cohort. Together, these data suggest that the levels of *LSD1* mRNA are higher in ovarian tumors than in normal tissue (with the likely exception of mucinous tumors).

The issue with this analysis is whether normal ovarian tissue is the best (or even a permissible) reference to establish abnormalities with regard to gene expression levels in ovarian tumors. Ovarian normal tissue is largely constituted of stromal cells, whereas ovarian tumors mainly derive from epithelial cells [40]. Although the distinction between stromal and epithelial cells is not always obvious at molecular level (for example, well-established tumors sometimes display a robust stromal gene signature even in the absence of visually apparent stroma [41]), it is obvious that a difference in cell type composition *per se* may cause differences in gene expression that might not be attributable to disease. On a different note, multiple studies suggest that ovarian tumors may derive from the fimbria of the fallopian tube, thus ovarian normal epithelium might not be a good reference control [40,42]. One known workaround to the use of normal (either ovarian or fallopian) tissue is to use cultured primary or immortalized normal epithelial cells. However, it is known that normal cells may quickly acquire aberrant features once in culture (reported in different systems, e.g. [43-45]), those expression differences not being associated with disease. Therefore, any reference control seems to be problematic in one or another way [46], as it might also be in our case (Figure 2A). Nevertheless, we suspect, that these aspects might be less relevant in the particular case of *LSD1*, because this gene is highly expressed in many cell and tissue types apparently without a strong cell/tissue-specific pattern of expression (i.e. being a housekeeping-like gene). Perhaps in agreement with this comment is the observation that *LSD1* mRNA levels are very similar in a comparison of borderline tumors (which are largely epithelial), different tumors at different stages (epithelial), mucinous tumors, and normal tissue (stromal). We show these comparisons later in this study.

In any case, to potentially avoid issues regarding the different origin of normal and tumor specimens, we subdivided the TCGA and our study cohort by FIGO stage, which allows us direct comparisons between tumor groups. In the TCGA cohort, we observed higher average levels of *LSD1* mRNA in stage II, III, and IV than in stage I tumors (Additional file 4: Figure S2A), but these differences did not reach significance likely because of the small number of stage I specimens in the TCGA cohort (n = 16). The differences (specially for stage III and IV) reached significance when compared to the larger group of normal tissue (n = 47), which shows similar average levels of *LSD1* mRNA to stage I tumors (Additional file 4: Figure S2A). In our cohort (in which, from now on, the few tumors of the mucinous subtype were removed from analysis), we could see statistically significant higher levels of *LSD1* mRNA in stage III tumors than in control and stage I tumors, although only after the application of a less stringent pair-comparison test (*p* = 0.0447, Mann–Whitney), but not after the application of a more stringent multi-comparison test (Kruskal-Wallis followed *post hoc* by Dunn’s, Figure 2B). Therefore, we suspect that the small size and high dispersion of tumor groups such as stage I diminish the power of our statistical analyses. Together, these analyses suggest moderately higher levels of *LSD1* mRNA in at least stage III ovarian tumors. The differences were robust compared to normal tissue, but not as robust in the direct comparison to stage I tumors.

We further subdivided stage III tumors into (sub)stages IIIA, IIIB, and IIIC. In the TCGA cohort, stage IIIC specimens showed the higher average levels of *LSD1* mRNA of the three (sub)stages, and the difference compared to control reached statistical significance (*p* < 0.0001 with restrictive Kruskal-Wallis/Dunn’s test; Additional file 4: Figure S2B). In our cohort, we observed a similar result (*p* = 0.0003, Kruskal-Wallis/Dunn’s test; Figure 2C). We also observed a statistically significant difference between stage IIIC tumors and tumors classified as stage IIIA or IIIB, although only after these two groups were combined (IIIA/IIIB; *p* = 0.0400, Mann-Whitney test; Figure 2C), thus reinforcing the argument that small group sizes limit the power of our statistical analyses. These results suggest that *LSD1* mRNA levels are high in stage IIIC ovarian tumors.

Next, we subclassified tumors by FIGO grade (borderline malignant, highly differentiated or grade G1, moderately differentiated or grade G2, and low differentiated/undifferentiated or grade G3). In the TCGA cohort (which lacks borderline tumors), we observed higher levels of *LSD1* mRNA in G2 and G3 than in G1 tumors by multi-comparison analysis (*p* = 0.0085 and *p* = 0.0071, Kruskal-Wallis/Dunn’s test; Additional file 4: Figure S2C). In our cohort, we observed very similar average levels and dispersion of *LSD1* expression in normal ovarian tissue and borderline tumors, which are mainly epithelial in origin (Figure 2D). As in the TCGA cohort, we also observed the highest levels of *LSD1* mRNA in G2, and the difference in this case with respect to G3 tumors reached significance (*p* = 0.0467 by Mann–Whitney, Figure 2D). These results suggest that *LSD1* mRNA levels are abnormally high in at least grade G2 tumors, a trend that was similarly observed in both independent cohorts.

**Figure 3:**
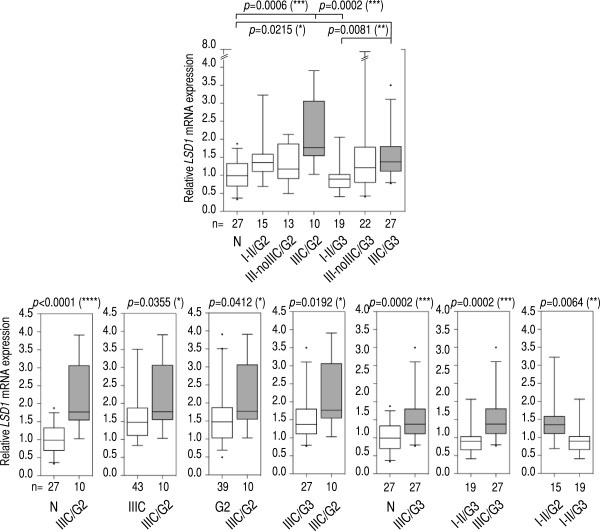
**Multi**- **and pair**-**comparison statistical tests suggest that a combination of stage IIIC and grade G2 is the diagnosis associated with the highest levels of *LSD1 *mRNA overexpression in our cohort.** Top panel: multiple-comparison analysis of normal tissue (N) and tumors subclassified as stage I or II and grade G2 (I-II/G2) or as stage I or II and grade G3 (I-II/G3); as stage III excluding IIIC and grade G2 (III-noIIIC/G2) or as stage III excluding IIIC and grade G3 (III-noIIIC/G3); and as stage IIIC and grade G2 (III/G2) or as stage IIIC and grade G3 (III/G3). Bottom panels: pair-comparison analyses in normal tissue (N), and stage IIIC and grade G2 (IIIC/G2) tumors (first panel); stage IIIC tumors, and stage IIIC and grade G2 (IIIC/G2) tumors (second panel); grade G2 tumors, and stage IIIC and grade G2 (IIIC/G2) tumors (third panel); stage IIIC and grade G3 (IIIC/G3) tumors, and stage IIIC and grade G2 (IIIC/G2) tumors (fourth panel); normal tissue (N), and stage IIIC and grade G3 (IIIC/G3) tumors (fifth panel); stage I or II and grade G3 (I-II/G3) tumors, and stage IIIC and grade G3 (IIIC/G3) tumors (sixth panel); and stage I or II and grade G2 (I-II/G2) tumors, and stage I or II and grade G3 (I-II/G3) tumors (seventh panel). We applied the Mann–Whitney test in pair-comparison analyses, and the Kruskal-Wallis (non-parametric ANOVA) test followed by *post hoc* Dunn’s analysis in multiple-comparison analyses (number of comparisons = 21). *P*-values are shown on top of each panel when significant. Number of specimens in each group is shown at the bottom of each panel. All histological subtypes were analyzed, excluding mucinous. Whiskers in box plots represent 5–95 percentile values, and horizontal lines within boxes represent median values. *P*-value < 0.05 (*), *p*-value < 0.01 (**), *p*-value < 0.001 (***), *p*-value < 0.0001 (****).

Finally, we combined FIGO stage and grade classifications for further analysis. In the TCGA cohort, we observed that tumors simultaneously classified as stage IIIC and grade G2 (IIIC/G2), IIIC/G3, or IV/G3 showed significantly higher levels of *LSD1* mRNA than control (even with stringent Kruskal-Wallis/Dunn’s analysis; Additional file 5: Figure S3). We observed a similar result in our cohort (Figure 3; although not with stage IV). Furthermore, IIIC/G2 and IIIC/G3 tumors showed significant higher *LSD1* levels than I-II/G3 tumors (*p* = 0.0002 and *p* = 0.0081, respectively, Kruskal-Wallis/Dunn’s; Figure 3). We also detected *LSD1* mRNA overexpression in IIIC/G2 specimens compared to stage IIIC or grade G2 tumors individually (*p* = 0.0355 and *p* = 0.0412, respectively, Mann–Whitney; Figure 3). IIIC/G2 tumors showed higher levels of *LSD1* mRNA than IIIC/G3 tumors (*p* = 0.0192, Mann–Whitney; Figure 3), or than any other tumor subgroup (e.g.: III-noIIIC/G2, *p* = 0.0303; III-noIIIC/G3, *p* = 0.0278; I-II/G2, *p* = 0.0066; or I-II/G3, *p* < 0.0001; Mann–Whitney). In contrast, III-noIIIC/G2 tumors (those classified as stage III excluding stage IIIC) were not statistically different than normal tissue. For IIIC/G3 specimens, we observed higher *LSD1* mRNA levels than in normal control or I-II/G3 tumors (*p* = 0.0002 and *p* = 0.0002, respectively, Mann–Whitney; Figure 3), but not than in stage IIIC or grade G3 tumors (analyzed independently), or than any other tumor group. I-II/G2 tumors also showed more *LSD1* mRNA than I-II/G3 tumors (*p* = 0.0064, Mann–Whitney; Figure 3). Together, these results suggest that *LSD1* mRNA overexpression in ovarian tumors reaches its highest level when they are simultaneously classified as stage IIIC and grade G2 or G3, independently of the cohort.

Overall, our analyses of *LSD1* mRNA levels in ovarian tumors suggest moderate *LSD1* mRNA overexpression in certain FIGO stages and grades, often observed consistently in two independent cohorts. In particular, we observed *LSD1* mRNA overexpression in stage IIIC tumors, as well as associated with high-grade tumors (G2 and, to a certain point, G3). In the TCGA cohort, we also observed *LSD1* mRNA upregulation in stage II and IV tumors, which may have been missed in our cohort due to the limited size of these two particular groups (n = 14 and n = 10 in our cohort, respectively, versus n = 27 and n = 85 in the TCGA cohort). Inconsistencies between cohorts might be attributed to disparities in tumor collections (sample size, donors, and composition of tumor subtypes), and to the use of different profiling techniques (qRT-PCR and microarray). In any case, we admit that the strength of our conclusions will strongly depend on the suitability of using normal tissue as reference control in the study of *LSD1* mRNA levels.

### Transcriptomic features of high *LSD1* mRNA-expressing ovarian tumors

**Figure 4:**
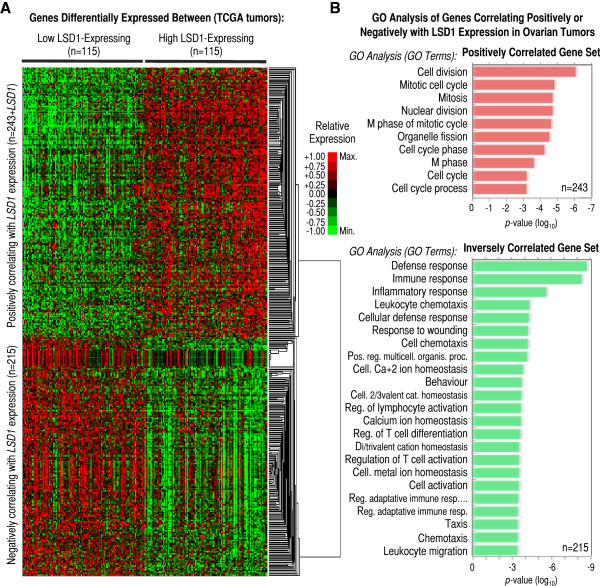
**Differential expression of genes involved in cell cycle and the immune/inflammatory response associated with *LSD1 *mRNA overexpression in the TCGA ovarian cohort. (A)** Heatmap showing hierarchical gene clustering analysis of expression profiles for n = 243 genes positively and n = 215 genes negatively correlating with *LSD1* mRNA expression in n = 115 low and n = 115 high LSD1-expressing ovarian tumors (source: TCGA). Genes positively correlating with *LSD1* are in the top-half section of the heatmap, while genes negatively correlation with *LSD1* at the bottom-half section after clustering. Genes in this analysis were included based on: 1) showing differential expression (fold-change) >1.5 for overexpressed or <1/1.5 for underexpressed genes between the two sets of high and low *LSD1*-expressing tumors; 2) reaching a Delta = 3 in the comparison between both tumors sets by SAM analysis, which corresponds to FDR = 3e-05; and, 3) correlating with *LSD1* expression in at least 50% tumors (both directions). The values of expression for each gene were independently normalized between the maximum (+1, or bright red) and the minimum expression levels detected (−1, or bright green) for the same particular gene (see legend), which does not allow absolute (but only relative) quantitative comparisons among genes. Therefore, overexpression is shown in red and underexpression in green. **(B)** List of most-enriched gene ontology (GO) terms in the set of n = 243 genes positively (top panel, red) and n = 215 genes negatively or inversely (bottom panel, green) correlating with the pattern of *LSD1* mRNA expression. GO terms shown in the left. The x-axis refers to *p*-value of enrichment.

Once we have identified that some ovarian tumors may show abnormally high levels of *LSD1* mRNA, we sought to explore whether these tumors may exhibit other molecular features. LSD1 (via its demethylase activity) is known to act as a positive and negative regulator of gene transcription [1,7,8,10,47]. Its overexpression, therefore (if correlated with overexpression of LSD1 protein and, especially, with enhanced LSD1 activity), might lead to additional transcriptomic alterations. It might also be possible that the same mechanisms leading to changes in *LSD1* gene expression may cause other transcriptomic alterations simultaneously. Either way, it is likely that an increase in the levels of *LSD1* mRNA is not an isolated event and correlates with other transcriptomic alterations. To identify these other potential alterations, we ranked TCGA ovarian tumors (n = 573) by levels of *LSD1* mRNA, and selected those cases showing *LSD1* mRNA overexpression or underexpression compared to the rest of tumors in the dataset (see Methods for more details). Both groups contained the same number of specimens, n = 115. By SAM analysis, we identified n = 821 (plus *LSD1*) genes that were differentially expressed between the two groups with fold change of at least 1.5 (Delta = 3, false discovery rate or FDR = 3e-05; Additional file 6: Figure S4A and S4B): n = 430 + *LSD1* overexpressed (fold change > 1.5), and n = 391 underexpressed (fold change < 1/1.5). Since some of these genes with differential expression concentrated these changes in a relatively small number of specimens (e.g. genes in the orange dotted-line box in Additional file 6: Figure S4B), we further filtered the full gene set to identify those cases with more consistent alterations (i.e. those that correlated with changes in *LSD1* mRNA expression in more than 50% of high *LSD1*-expressing and 50% of low *LSD1*-expressing tumors; Additional file 6: Figure S4C). This filter reduced the number of differentially expressed genes to n = 458 (plus *LSD1*): n = 243 genes positively correlating with *LSD1* expression, and n = 215 cases negatively correlating with *LSD1* expression (Figure 4A and Additional file 2: Table S2). The following are examples of those genes that clustered the most with *LSD1* in this analysis (positive correlation): *FRAP1*/*mTOR*, *CDC2L2*, *CCDC21*, *PINK1*, *KIAA0495*, *NOC2L*/*NIR*, *SLC25A33*, *NECAP2*, *KIAA0090*, *EN01*, *LUZP1*, *CAPZB*, *DNAJC16*, *PGD*, *C1orf128*, *LYPLA2*, *UBE2J2*, *LRRC47*, *DNAJC11*, and *WDR8*.

To functionally characterize the filtered gene set, we next performed gene ontology (GO) analysis finding that the group of genes positively correlated with *LSD1* (i.e. those overexpressed in high *LSD1*-expressing tumors and underexpressed in low *LSD1*-expressing tumors) is enriched in GO terms of cell cycle and mitosis (Figure 4B, top). Interestingly, LSD1 is known to control cell proliferation in cancer cells [48]. On the other hand, GO analysis of the group of genes that showed a pattern of expression that negatively correlated with that of *LSD1* (i.e. those underexpressed in high *LSD1*-expressing tumors and overexpressed in low *LSD1*-expressing tumors) showed enrichment in GO terms related with the defense/immune/inflammatory response (Figure 4B, bottom). This last observation is of special interest due to the importance of the immune response in the pathology of ovarian cancer, particularly in contributing to the pattern of T-cell infiltration in ovarian tumors [49,50]. In fact, a set of high-grade serous ovarian tumors (known as subtype C5) has been recently defined by the simultaneous overexpression of cell proliferation genes and underexpression of an immune signature (among other features), and it is clinically associated with poor overall survival [41]. Based on this report, we also examined whether high *LSD1*-expressing tumors are associated with poor overall patient survival, but failed to find such association with our set of n = 115 high *LSD1*-expressing tumors (Additional file: 6 Figure S4D). In contrast, we found, using the cBioPortal for Cancer Genomic [51], poor patient survival and reduced disease free survival associated with high *LSD1*-expressing tumors (fold-change > 1.20, n = 30) in the cohort of n = 580 TCGA specimens (log-rank test *p* = 0.002644 and *p* = 0.012440, respectively; Additional file 7: Figure S5). Together, we have identified a rich molecular signature of differentially expressed cell cycle and immune/inflammatory genes associated with *LSD1* mRNA overexpression in ovarian tumors. Whether this molecular (transcriptomic) signature is functionally (biologically) relevant and, if so, whether it results directly from alterations in *LSD1* expression or instead is induced by the same mechanisms that alter *LSD1* expression is yet to be determined.

### Chemical inhibitors of LSD1 activity are cytotoxic for ovarian cancer cells

**Figure 5:**
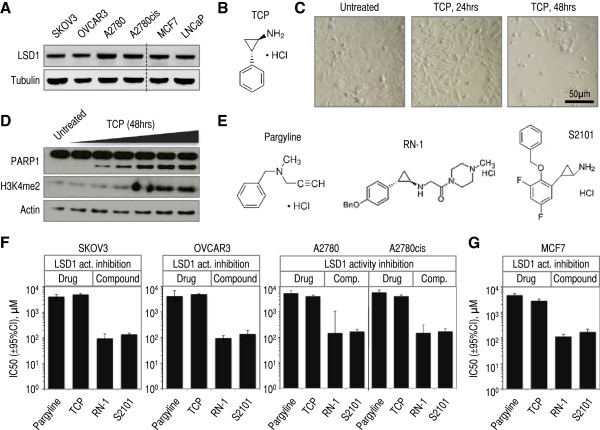
**Chemical LSD1 inhibitors reduce the cell viability of a panel of ovarian cancer cell lines. (A)** Western blot analysis of LSD1 protein levels in a panel of human ovarian cancer lines (SKOV3, OVCAR-3, A2780, and the cisplatin-tolerant derivative A2780cis). We show also analysis of LSD1 protein expression in two positive controls: breast cancer MCF7 cells and prostate cancer LNCaP cells. **(B)** Chemical structure of non-selective LSD1 inhibitor tranylcypromine (TCP or 2-PCPA). **(C)** Representative bright-field microscopy images of SKOV3 cells treated with LSD1 inhibitor TCP for the time labeled on top of the panels. Magnification x10. Bar = 50 μm. **(D)** Western blot analysis of PARP1 cleavage (note: cleaved fragment corresponds to the band of faster migration), and LSD1 substrate H3K4me2 in SKOV3 cell treated with increasing amounts of LSD1 inhibitor TCP. **(E)** Chemical structure of non-selective LSD1 inhibitors: pargyline, RN-1, and S2101. **(F)** Estimated IC50 values and 95% CI for each LSD1 inhibitor in our panel of ovarian cancer lines (extracted from the graphs shown in Additional file 8: Figure S6B-S6E). The y-axis is log-10 scale. **(G)** Estimated IC50 values and 95% CI for each LSD1 inhibitor in breast cancer MCF7 cells. The y-axis is log-10 scale.

Finally, chemical inhibition of LSD1 is emerging as having potential therapeutic value in the treatment of some cancer types and blood disorders [48,52]. Our results indicating that the levels of *LSD1* mRNA might be aberrant in some ovarian tumors, and that they might also be linked to upregulation of cell cycle genes and downregulation of genes of the immune/inflammatory response prompted us to initiate studies to test the potential cytotoxic effects of LSD1 inhibitors in a panel of ovarian cancer cells (SKOV3, A2780 and OVCAR3, and the cisplatin resistant clone A2780cis, which derives from A2780 cells). First, we confirmed LSD1 protein expression in all these lines (using breast cancer MCF7 cells [8] and prostate cancer LNCaP cells [7] as reference, Figure 5A). Next, we treated SKOV3 cells with arguably the most studied LSD1 inhibitor: TCP [30-33,53-55] (Figure 5B). It was immediately apparent that TCP induced death of SKOV3 cells, because this treatment decreased abruptly the number of cells (Figure 5C). Likely this effect resulted from TCP-induced apoptosis, because we observed a gradual increase in the levels of PARP1 cleavage associated with the treatment (Figure 5D, PARP1). This effect correlated with the expected gradual increase in the levels of LSD1 substrate H3K4me2 [1], which confirms the LSD1 inhibitory effect of the treatment (Figure 5D, H3K4me2). To evaluate cell death in the full panel of ovarian cancer lines, and to compare the efficacy of different LSD1 inhibitors to decrease cell viability in these lines, we tested TCP (FDA-approved drug), pargyline (FDA-approved drug), and more potent and selective RN-1 and S2101 compounds (Figure 5E), as well as also potent and selective polyamine analog CAS 927019-63-4 and amidino-guanidinium CBB1007 compounds (Additional file 8: Figure S6A). All these compounds are reported LSD1 inhibitors. These tests were performed by the MTS/PMS cell viability assay after 48 hours drug/compound treatment. Four inhibitors induced clear cytotoxicity in all four cell lines: pargyline and TCP at millimolar concentrations (Additional file 8: Figure S6B-S6E, red and orange lines, respectively), and RN-1 and S2101 at micromolar concentrations (Additional file 8: Figure S6B-S6E, dark and light blue lines, respectively). CBB1007 induced cytotoxicity but the same effect was observed in a parallel treatment with vehicle DMSO (see Methods; data not shown). CAS 927019-63-4 did not induce cytotoxicity at any of the tested concentrations, for which it was also excluded from our panel (data not shown). For pargyline, TCP, RN-1 and S2101, we estimated their half maximal inhibitory concentrations (IC50), which roughly correlated with their reported potency in inhibiting LSD1: potent RN-1 and S2101 showed lower IC50 values, and weaker pargyline and TCP showed higher IC50 values (Figure 5F, log10-scale). Importantly, RN-1 and S2101 are also known to be more selective than pargyline and TCP for LSD1, which has value in a potential therapeutic use of these compounds. For comparison, we also estimated IC50s for the same inhibitors in breast cancer MCF7 cells observing values in the same range (compare Figure 5G). Together, our data suggest that LSD1 inhibitors are cytotoxic agents in ovarian cancer cells, although they show relatively mild effects when compared to cisplatin in parallel treatments (RN-1 and S2101 IC50 values ≈100-200 μM, while cisplatin ≈5-20 μM, depending of the cell line; data not shown).

## Conclusions

LSD1 is a lysine demethylase whose activity is involved in cancer biology, but a link to ovarian cancer has not yet been directly explored. Histone lysine methylation and demethylation have gained significant attention since their discovery [1,56]. These two antagonistic activities are emerging as important in cancer biology via regulating histone and non-histone substrates [57]. The list of methylated proteins is continuously expanding [58], and the field seems to be flourishing with intriguing new data. Here, we have studied LSD1 in the context of ovarian tumors and cancer cells. Our studies suggest the moderate overexpression of *LSD1* mRNA in stage IIIC and high-grade ovarian tumors. The lack of a strong association between the levels of *LSD1* mRNA and the disease could be due to the fact that we limited our analyses to the measurement of the transcript. This association could be stronger (or weaker or even absent) at the level of the functional entities: the LSD1 protein and its activity. Future studies, therefore, will be required to tests these associations. That said, LSD1 might be important in ovarian cancer independently whether its levels/activity change. In breast and prostate cancer cells, for example, LSD1 is required for the stimulation of the hormonal signaling response and cell proliferation [7,8]. In fact, our observation that the four ovarian cancer lines tested in this study show robust levels of LSD1 (protein) expression may suggest a functional role of LSD1 also in these cells.

At molecular level, our study suggests that tumors showing high levels of *LSD1* mRNA also exhibit a signature of relative overexpression of genes involved in cell cycle and underexpression of genes involved in the immune response, which in the past has been associated to aggressive tumors in the context of other cohorts [41]. Perhaps in agreement, we found a set of high *LSD1* mRNA-expressing tumors associated with poor patient survival (Additional file 7: Figure S5), although we failed to observe the same association with the full set of tumors that we analyzed to derive the mentioned signature (Additional file 6: Figure S4D). We also report the cytotoxic effects of a panel of chemical LSD1 inhibitors in a panel of ovarian cancer cell lines. Additional studies will be necessary to determine the robustness of these inhibitory effects *in vivo*, as well as their secondary effects, before we can consider these compounds as potential therapies for the disease. Together, our study prompts further exploration of LSD1 and the LSD1 demethylase activity in ovarian cancer and their targeting for potential therapeutic purposes.

## Competing interest

The authors declare that they have no competing interest.

## Authors’ contributions

SK analyzed and interpreted data, and contributed to the writing of the manuscript. IGB conceived and designed the study, performed experiments, analyzed and interpreted data, and wrote the manuscript. Both authors have read and approved the final manuscript.

## Supplementary Material

Additional file 1: Table S1 Summary of clinicopathologic features of the study cohort. This summary includes patient age and gender, specimen tissue origin and body localization, diagnosis, histological subtype, and tumor FIGO stage and grade. Click here for file

Additional file 2: Table S2 List of differentially expressed genes in high and low *LSD1*-expressing tumors in the TCGA cohort. Criteria for selection: fold-change >1.5 or <1/1.5, Delta = 3, FDR < 3e-05, and expression correlation with *LSD1* in at least 50% tumors. Click here for file

Additional file 3: Figure S1 Pair-comparison statistical tests suggest mucinous ovarian tumors as the only histological subtype not showing *LSD1* mRNA overexpression in our study cohort. For a more faithful comparison between histological subtypes, we compared only tumors classified as adenocarcinoma in each subtype and also excluded those specimens in which more than one histological subtype was detected (final number of tumor specimens in this analysis n = 109). Top panel: multi-comparison analysis. Rest of panels: pair-comparison analyses. Ovarian normal tissue (N), serous (S), papillary serous (PS), endometrioid (E), clear cell (CC), and mucinous (M). Measured by qRT-PCR in our cohort (values expressed as relative to normal average). We applied the Mann–Whitney test in pair-comparison analyses, and the Kruskal-Wallis (non-parametric ANOVA) test followed by *post hoc* Dunn’s analysis in multiple-comparison analyses. *P*-values are shown on top of each panel when significant (in two cases, *p*-values of 0.0556 and 0.0514 were also indicated despite not reaching significance). Number of specimens in each group is shown at the bottom of each panel. Whiskers in box plots represent 5–95 percentile values, and horizontal lines within boxes represent median values. *P*-value < 0.05 (*), *p*-value < 0.01 (**), *p*-value < 0.001 (***), *p*-value < 0.0001 (****). Click here for file

Additional file 4: Figure S2 Multi- and pair-comparison statistical tests suggest *LSD1* mRNA overexpression in stage IIIC and grade G2/G3 (and other) specimens in the TCGA cohort. **(A)** Left panel: multi-comparison analysis of normal tissue (N) and tumors subclassified as stage I-IV (I-IV). Rest of panels: pair-comparison analyses of normal tissue (N) and stage II (II) tumors (left); normal tissue (N) and stage III (III) tumors (middle); or normal tissue (N) and stage IV (IV) tumors (right). **(B)** Left panel: multiple-comparison analysis of normal tissue (N) and tumors subclassified as stage III, IIIB, or IIIC (IIIA-IIIC). Middle and right panels: pair-comparison analysis of normal tissue (N) and stage IIIA (IIIA) or stage IIIC tumors (IIIC), respectively. **(C)** Left panel: multiple-comparison analysis of normal tissue (N) and tumors subclassified as grade G1, grade G2, or grade G3 (G1-G3). Rest of panels: pair-comparison analyses of normal tissue (N) and grade G1 (G1) tumors (first panel); normal tissue (N) and grade G2 (G2) tumors (second panel); normal tissue (N) and grade G3 (G3) tumors (third panel); grade G1 (G1) and grade G2 (G2) tumors (fourth panel); and grade G1 (G1) and grade G3 (G3) tumors (fifth panel). Measured by microarray in TCGA cohort (log-2 scale). TCGA tumors belong only to the serous cystadenocarcinoma subtype. We applied the Mann–Whitney test in pair-comparison analyses, and the Kruskal-Wallis (non-parametric ANOVA) test followed by *post hoc* Dunn’s analysis in multiple-comparison analyses. *P*-values are shown on top of each panel when reach significance. Number of specimens in each analyzed group is shown at the bottom of each panel. Y-axis is log2 scale. Whiskers in box plots represent 5–95 percentile values, and horizontal lines within boxes represent median values. *P*-value < 0.05 (*), *p*-value < 0.01 (**), *p*-value < 0.001 (***), *p*-value < 0.0001 (****). Click here for file

Additional file 5: Figure S3 Multi- and pair-comparison statistical tests suggest the highest levels of *LSD1* mRNA overexpression in ovarian tumors to be associated with a combination of stage IIIC and grade G2 or G3 tumor (or stage IV and grade G3) features in the TCGA cohort. Left panel: multiple-comparison analysis of normal tissue (N) and tumors simultaneously subclassified as stage I or II and grade G2 (I-II/G2), stage I or II and grade G3 (I-II/G3), stage III excluding IIIC and grade G2 (III-noIIIC/G2), stage III excluding IIIC and grade G3 (III-noIIIC/G3), stage IIIC and grade G2 (III/G2), stage IIIC and grade G3 (III/G3), stage IV and grade G2 (IV/G2), and stage IV and grade G3 (IV/G3). Rest of panels: pair-comparison analyses of normal tissue (N) and stage IIIC and grade G2 (IIIC/G2) tumors (left), normal tissue (N) and stage IIIC and grade G3 (IIIC/G3) tumors (middle), and normal tissue (N) and stage IV and grade G3 (IV/G3) tumors (right). Measured by microarray in TCGA cohort (log-2 scale). TCGA tumors belong only to the serous cystadenocarcinoma subtype. To detect differences between groups in each panel, we applied the Mann–Whitney test in pair-comparison analyses, and the Kruskal-Wallis (non-parametric ANOVA) test followed by *post hoc* Dunn’s analysis in multiple-comparison analyses. Number of comparisons = 10 (in A) and 6 (in B and C). *P*-values are shown on top of each panel when reach significance. Number of specimens in each analyzed group is shown at the bottom of each panel. Whiskers in box plots represent 5–95 percentile values, and horizontal lines within boxes represent median values. *P*-value < 0.05 (*), *p*-value < 0.01 (**), *p*-value < 0.001 (***), *p*-value < 0.0001 (****). Click here for file

Additional file 6: Figure S4 Differential transcriptomic profiles and patient survival associated with high and low *LSD1*-expressing tumors in the TCGA cohort. **(A)** SAM analysis of genes showing differential expression between the sets of n = 115 high and n = 115 low *LSD1*-expressing ovarian tumors (source: TCGA). Green circles indicate genes with differential expression (n = 822 total; n = 431 overexpressed, on top, and n = 391 underexpressed, at the bottom). Delta = 3, which corresponds to FDR = 3e-05. **(B)** Heatmap analysis of hierarchically clustered expression profiles of the n = 822 genes showing differential expression between high and low *LSD1*-expressing tumors (source: TCGA). The values of expression for each gene were independently normalized between the maximum (+1, or bright red) and the minimum expression levels detected (−1, or bright green) for the same particular gene (see legend), which does not allow absolute (only relative) quantitative comparisons among genes. Therefore, overexpression is shown in red and underexpression in green. The orange line indicates a few representative examples of genes that show differential expression in a few only of tumors. **(C)** Number of genes positively or negatively (inversely) correlating with *LSD1* mRNA expression in high and low *LSD1*-expressing tumors based on the number of tumors in which this property is observed (>80%, >70%, >50%, >30%, or <30% of tumors). The total number of genes is also indicated. (**D**) Kaplan-Meier curve of overall survival associated with tumors classified based on low (n = 115), high (n = 115), and the rest (medium) *LSD1* expression. Log-rank (Mantel-Cox) *p*-value and number of samples are indicated. Click here for file

Additional file 7: Figure S5 Information associated with LSD1-overexpressing ovarian tumors in the cBioPortal for Cancer Genomics (based on a subset of the TCGA cohort). Oncoprint identifies n = 30 tumors in a cohort of n = 580 specimens showing *LSD1* (*KDM1A*) mRNA overexpression (cancer study: Ovarian Serous Cystadenocarcinoma TCGA-Provisional; genomic profiles: mRNA Expression z-Scores RNA Seq V2 RSEM and RPPA protein/phosphoprotein level z-score threshold ±2; patient/case set: all tumors; query: ‘KDM1A: EXPR > 1.20’). Query performed on October 7, 2013. Kaplan-Meier curve of overall survival (left) and disease free survival (right) associated with these n = 30 tumors. Log-rank (Mantel-Cox) *p*-value and number of samples are indicated. Protein levels and phosphorylation changes observed in the same set of n = 30 tumors. Panels extracted from the cBioPortal for Cancer Genomics (developed by the Computational Center at Memorial Sloan-Kettering Cancer Center and the i-Vis Research Group of the Computer Engineering Department at Bilkent University). Click here for file

Additional file 8: Figure S6 Analysis of cell viability upon treatment with chemical LSD1 inhibitors in a panel of ovarian cancer lines. **(A)** Chemical structures of LSD1 inhibitors CAS 927019-63-4 and CBB1007. **(B-E)** MTS/PMS viability assay in SKOV3, OVCAR-3, A2780, and A2780cis cells treated with different LSD1 inhibitors: pargyline (red), TCP (orange), RN-1 (light blue), and S2101 (dark blue). Measurements are shown as dots. Error bars represent s.e.m. Lines represent the estimated inhibitory curve response. The x-axis is log-10 scale. Click here for file
