# Supplementary material for: Analysis of the levels of lysine-specific demethylase 1 (LSD1) mRNA in human ovarian tumors and the effects of chemical LSD1 inhibitors in ovarian cancer cell lines
Source: J Ovarian Res. 2013 Oct 29;6:75. doi: 10.1186/1757-2215-6-75 (PMC4176291; doi:10.1186/1757-2215-6-75)
Supplement: Additional file 1: Table S1 — Summary of clinicopathologic features of the study cohort. This summary includes patient age and gender, specimen tissue origin and body localization, diagnosis, histological subtype, and tumor FIGO stage and grade. [file 1757-2215-6-75-S1.pdf]

Clinicopathologic parameters of the study cohort (n=177)

Gender: female

Age: 23–91 years (mean=57.67)

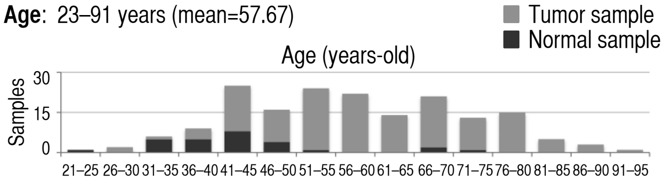

**Tissue origin:**		**Sample tissue source:**	
Ovary	n=177	Normal ovary	n=27
		Tumor, ovary	n=128
		Tumor, metastatic site*	n=22

**Diagnosis**			
Adenocarcinoma	n=123	Carcinoma	n=17
Serous	n=38	Papillary serous	n=8
Papillary serous	n=39	Endometrioid	n=6
Endometrioid	n=29	Clear cell	n=1
Clear cell	n=7	Other	n=2
Mucinous	n=5	Carcinosarcoma	n=1
Other	n=5	Tumor, borderline	n=9

**FIGO stage classification**:			
Stage 0 (normal)	n=27	Stage IIIA	n=13
Stage IA	n=19	Stage IIIB	n=22
Stage IB	n=9	Stage IIIC	n=44
Stage IC	n=11	Stage IV	n=10
Stage II, unc.	n=1	**FIGO tumor grade classification**:	
Stage IIA	n=2	Borderline malignancy	n=7
Stage IIB	n=9	G1	n=12
Stage IIC	n=3	G2	n=41
Stage III, unc.	n=7	G3	n=74
		Not Reported	n=16

* Omentum, peritoneum, lymph node, pelvic wall, liver, or colon.
