# Supplementary figures and images for: Analysis of the levels of lysine-specific demethylase 1 (LSD1) mRNA in human ovarian tumors and the effects of chemical LSD1 inhibitors in ovarian cancer cell lines
Source: J Ovarian Res. 2013 Oct 29;6:75. doi: 10.1186/1757-2215-6-75 (PMC4176291; doi:10.1186/1757-2215-6-75)

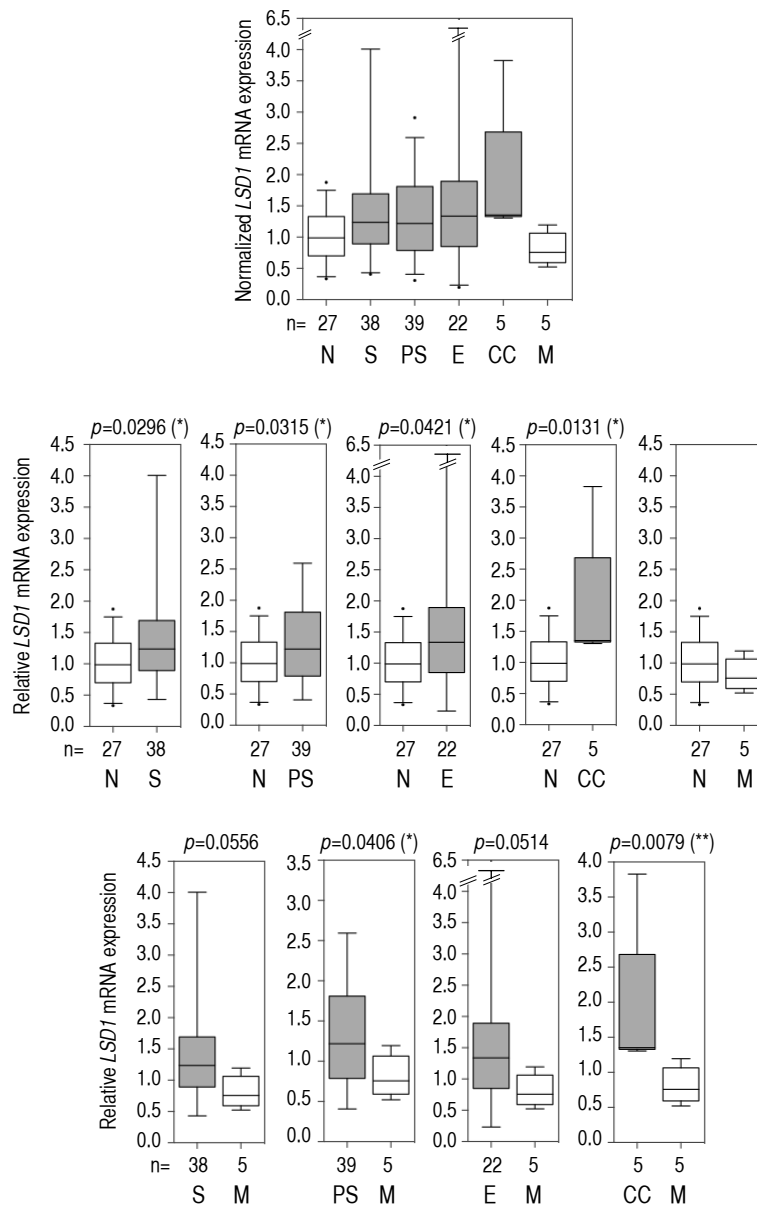

Supplement: Additional file 3: Figure S1 — Pair-comparison statistical tests suggest mucinous ovarian tumors as the only histological subtype not showing LSD1 mRNA overexpression in our study cohort. For a more faithful comparison between histological subtypes, we compared only tumors classified as adenocarcinoma in each subtype and also excluded those specimens in which more than one histological subtype was detected (final number of tumor specimens in this analysis n = 109). Top panel: multi-comparison analysis. Rest of panels: pair-comparison analyses. Ovarian normal tissue (N), serous (S), papillary serous (PS), endometrioid (E), clear cell (CC), and mucinous (M). Measured by qRT-PCR in our cohort (values expressed as relative to normal average). We applied the Mann–Whitney test in pair-comparison analyses, and the Kruskal-Wallis (non-parametric ANOVA) test followed by post hoc Dunn’s analysis in multiple-comparison analyses. P-values are shown on top of each panel when significant (in two cases, p-values of 0.0556 and 0.0514 were also indicated despite not reaching significance). Number of specimens in each group is shown at the bottom of each panel. Whiskers in box plots represent 5–95 percentile values, and horizontal lines within boxes represent median values. P-value < 0.05 (*), p-value < 0.01 (**), p-value < 0.001 (***), p-value < 0.0001 (****). [file 1757-2215-6-75-S3.pdf]

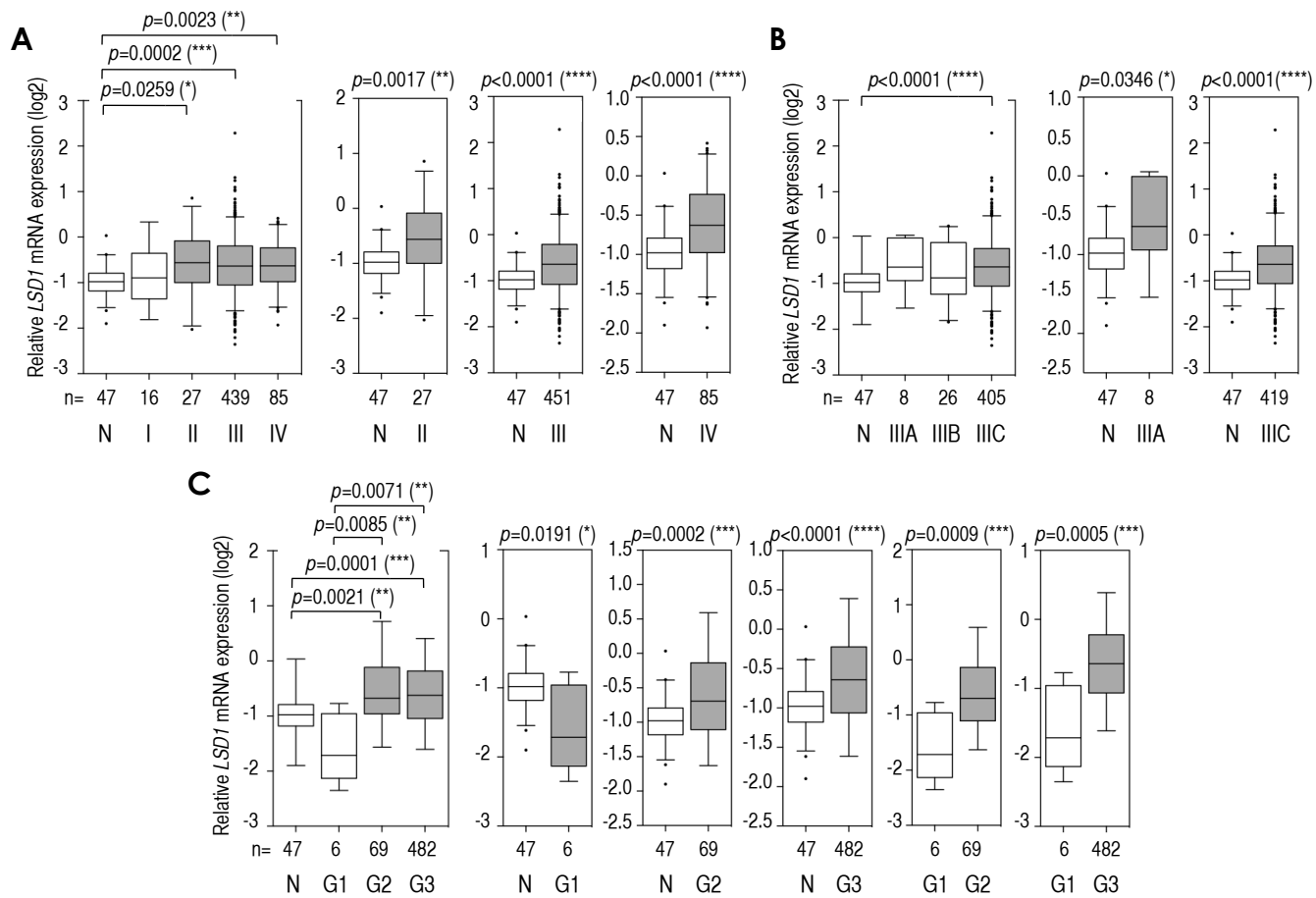

Supplement: Additional file 4: Figure S2 — Multi- and pair-comparison statistical tests suggest LSD1 mRNA overexpression in stage IIIC and grade G2/G3 (and other) specimens in the TCGA cohort. (A) Left panel: multi-comparison analysis of normal tissue (N) and tumors subclassified as stage I-IV (I-IV). Rest of panels: pair-comparison analyses of normal tissue (N) and stage II (II) tumors (left); normal tissue (N) and stage III (III) tumors (middle); or normal tissue (N) and stage IV (IV) tumors (right). (B) Left panel: multiple-comparison analysis of normal tissue (N) and tumors subclassified as stage III, IIIB, or IIIC (IIIA-IIIC). Middle and right panels: pair-comparison analysis of normal tissue (N) and stage IIIA (IIIA) or stage IIIC tumors (IIIC), respectively. (C) Left panel: multiple-comparison analysis of normal tissue (N) and tumors subclassified as grade G1, grade G2, or grade G3 (G1-G3). Rest of panels: pair-comparison analyses of normal tissue (N) and grade G1 (G1) tumors (first panel); normal tissue (N) and grade G2 (G2) tumors (second panel); normal tissue (N) and grade G3 (G3) tumors (third panel); grade G1 (G1) and grade G2 (G2) tumors (fourth panel); and grade G1 (G1) and grade G3 (G3) tumors (fifth panel). Measured by microarray in TCGA cohort (log-2 scale). TCGA tumors belong only to the serous cystadenocarcinoma subtype. We applied the Mann–Whitney test in pair-comparison analyses, and the Kruskal-Wallis (non-parametric ANOVA) test followed by post hoc Dunn’s analysis in multiple-comparison analyses. P-values are shown on top of each panel when reach significance. Number of specimens in each analyzed group is shown at the bottom of each panel. Y-axis is log2 scale. Whiskers in box plots represent 5–95 percentile values, and horizontal lines within boxes represent median values. P-value < 0.05 (*), p-value < 0.01 (**), p-value < 0.001 (***), p-value < 0.0001 (****). [file 1757-2215-6-75-S4.pdf]

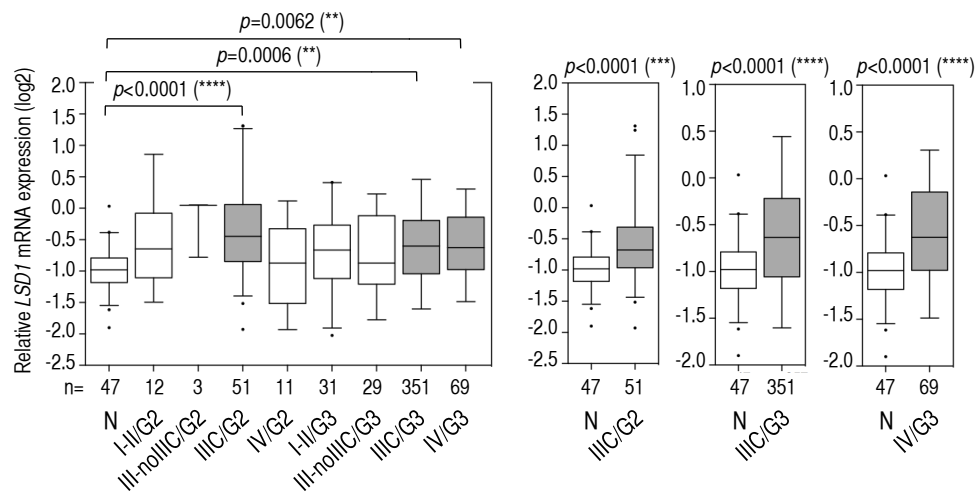

Supplement: Additional file 5: Figure S3 — Multi- and pair-comparison statistical tests suggest the highest levels of LSD1 mRNA overexpression in ovarian tumors to be associated with a combination of stage IIIC and grade G2 or G3 tumor (or stage IV and grade G3) features in the TCGA cohort. Left panel: multiple-comparison analysis of normal tissue (N) and tumors simultaneously subclassified as stage I or II and grade G2 (I-II/G2), stage I or II and grade G3 (I-II/G3), stage III excluding IIIC and grade G2 (III-noIIIC/G2), stage III excluding IIIC and grade G3 (III-noIIIC/G3), stage IIIC and grade G2 (III/G2), stage IIIC and grade G3 (III/G3), stage IV and grade G2 (IV/G2), and stage IV and grade G3 (IV/G3). Rest of panels: pair-comparison analyses of normal tissue (N) and stage IIIC and grade G2 (IIIC/G2) tumors (left), normal tissue (N) and stage IIIC and grade G3 (IIIC/G3) tumors (middle), and normal tissue (N) and stage IV and grade G3 (IV/G3) tumors (right). Measured by microarray in TCGA cohort (log-2 scale). TCGA tumors belong only to the serous cystadenocarcinoma subtype. To detect differences between groups in each panel, we applied the Mann–Whitney test in pair-comparison analyses, and the Kruskal-Wallis (non-parametric ANOVA) test followed by post hoc Dunn’s analysis in multiple-comparison analyses. Number of comparisons = 10 (in A) and 6 (in B and C). P-values are shown on top of each panel when reach significance. Number of specimens in each analyzed group is shown at the bottom of each panel. Whiskers in box plots represent 5–95 percentile values, and horizontal lines within boxes represent median values. P-value < 0.05 (*), p-value < 0.01 (**), p-value < 0.001 (***), p-value < 0.0001 (****). [file 1757-2215-6-75-S5.pdf]
