# Supplementary material for: Analysis of the levels of lysine-specific demethylase 1 (LSD1) mRNA in human ovarian tumors and the effects of chemical LSD1 inhibitors in ovarian cancer cell lines
Source: J Ovarian Res. 2013 Oct 29;6:75. doi: 10.1186/1757-2215-6-75 (PMC4176291; doi:10.1186/1757-2215-6-75)
Supplement: Additional file 6: Figure S4 — Differential transcriptomic profiles and patient survival associated with high and low LSD1-expressing tumors in the TCGA cohort. (A) SAM analysis of genes showing differential expression between the sets of n = 115 high and n = 115 low LSD1-expressing ovarian tumors (source: TCGA). Green circles indicate genes with differential expression (n = 822 total; n = 431 overexpressed, on top, and n = 391 underexpressed, at the bottom). Delta = 3, which corresponds to FDR = 3e-05. (B) Heatmap analysis of hierarchically clustered expression profiles of the n = 822 genes showing differential expression between high and low LSD1-expressing tumors (source: TCGA). The values of expression for each gene were independently normalized between the maximum (+1, or bright red) and the minimum expression levels detected (−1, or bright green) for the same particular gene (see legend), which does not allow absolute (only relative) quantitative comparisons among genes. Therefore, overexpression is shown in red and underexpression in green. The orange line indicates a few representative examples of genes that show differential expression in a few only of tumors. (C) Number of genes positively or negatively (inversely) correlating with LSD1 mRNA expression in high and low LSD1-expressing tumors based on the number of tumors in which this property is observed (>80%, >70%, >50%, >30%, or <30% of tumors). The total number of genes is also indicated. (D) Kaplan-Meier curve of overall survival associated with tumors classified based on low (n = 115), high (n = 115), and the rest (medium) LSD1 expression. Log-rank (Mantel-Cox) p-value and number of samples are indicated. [file 1757-2215-6-75-S6.pdf]

A

SAM Analysis (Delta = 3)

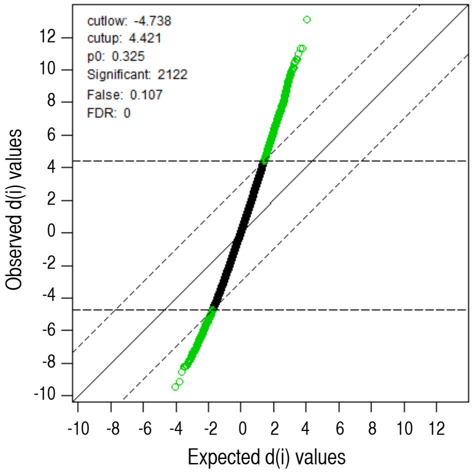

B

Genes Differentially Expressed Between:

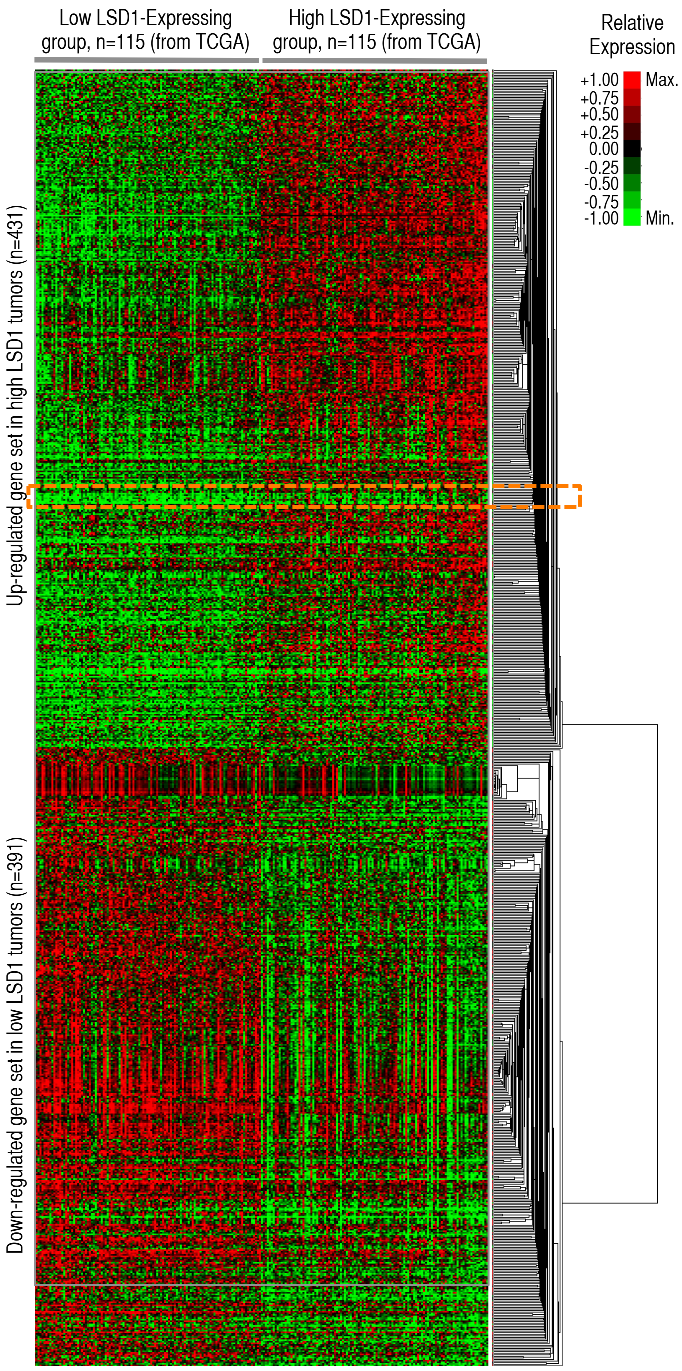

C

Genes showing expression correlation with *LSD1* in:

**Percentage of specimens**	Positively correlating	Negatively correlating	Total
>80%	4	0	4
>70%	35	2	37
>50%	243	215	458
>30%	382	346	728
<30%	430	391	821

D

Kaplan-Meier Analysis

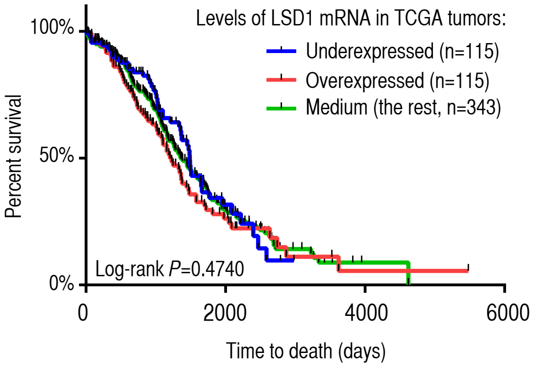

Konovalov and Garcia-Bassets, **Figure S4**
