# Supplementary material for: Analysis of the levels of lysine-specific demethylase 1 (LSD1) mRNA in human ovarian tumors and the effects of chemical LSD1 inhibitors in ovarian cancer cell lines
Source: J Ovarian Res. 2013 Oct 29;6:75. doi: 10.1186/1757-2215-6-75 (PMC4176291; doi:10.1186/1757-2215-6-75)
Supplement: Additional file 7: Figure S5 — Information associated with LSD1-overexpressing ovarian tumors in the cBioPortal for Cancer Genomics (based on a subset of the TCGA cohort). Oncoprint identifies n = 30 tumors in a cohort of n = 580 specimens showing LSD1 (KDM1A) mRNA overexpression (cancer study: Ovarian Serous Cystadenocarcinoma TCGA-Provisional; genomic profiles: mRNA Expression z-Scores RNA Seq V2 RSEM and RPPA protein/phosphoprotein level z-score threshold ±2; patient/case set: all tumors; query: ‘KDM1A: EXPR > 1.20’). Query performed on October 7, 2013. Kaplan-Meier curve of overall survival (left) and disease free survival (right) associated with these n = 30 tumors. Log-rank (Mantel-Cox) p-value and number of samples are indicated. Protein levels and phosphorylation changes observed in the same set of n = 30 tumors. Panels extracted from the cBioPortal for Cancer Genomics (developed by the Computational Center at Memorial Sloan-Kettering Cancer Center and the i-Vis Research Group of the Computer Engineering Department at Bilkent University). [file 1757-2215-6-75-S7.pdf]

Memorial Sloan-Kettering Cancer Center

cBioPortal for Cancer Genomics

Visualize, analyze, discover.

HOME | DATA SETS | WEB API | R/MATLAB | TUTORIALS | FAQ | NEWS | ABOUT

Gene Set / Pathway is altered in 5.2% of all cases.

Ovarian Serous Cystadenocarcinoma (TCGA, Provisional)/All Tumors: (580)/User-defined List/1 gene

OncoPrint

Case Set: All Tumors: All tumor samples (580 samples)

Altered in 30 (5%) of cases

KDM1A 5%

mRNA Upregulation

Copy number alterations are putative.

Overall Survival

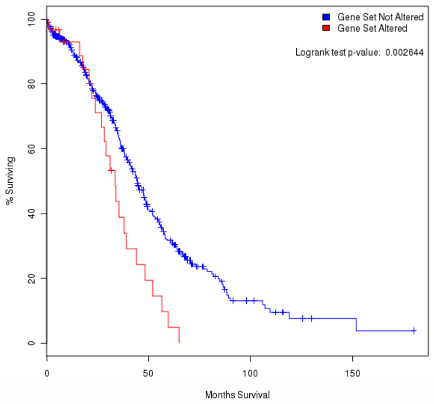

Disease Free Survival

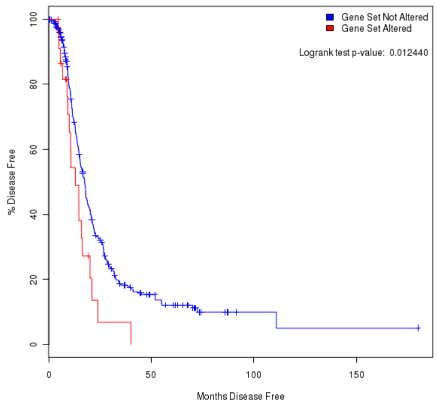

Protein Levels (top) and Phosphorylation (bottom) Changes

Antibody Type: protein_level   Search:

Target		Ave. Abundance		p-value	Plot
Protein	Residue	Unaltered	Altered		
TGM2		0.03	-0.48	1.49e-6 ▼	⊕
CASP7	cleavedD198	0.04	-0.59	4.59e-6 ▼	⊕
MSH2		-0.07	1.01	4.81e-6 ▲	⊕
LCK		0.06	-0.90	1.48e-5 ▼	⊕
MSH6		-0.07	1.06	1.79e-5 ▲	⊕
PTCH1		0.04	-0.71	4.08e-5 ▼	⊕
EGFR		0.04	-0.48	2.36e-4 ▼	⊕
RBM3		0.06	-0.79	2.64e-4 ▼	⊕
CCNB1		-0.05	0.69	2.70e-4 ▲	⊕
ANXA1		0.06	-0.80	4.66e-4 ▼	⊕
INPP4B		0.03	-0.53	6.67e-4 ▼	⊕
BAX		0.03	-0.60	8.77e-4 ▼	⊕
NF2		0.05	-0.77	0.001 ▼	⊕
IGFBP2		-0.04	0.55	0.003 ▲	⊕
PTGS2		0.04	-0.44	0.004 ▼	⊕
XIAP		0.03	-0.49	0.004 ▼	⊕
STMN1		-0.02	0.49	0.005 ▲	⊕
MAPK9		0.03	-0.54	0.008 ▼	⊕
BID		0.04	-0.49	0.008 ▼	⊕
CDK1		-0.03	0.55	0.008 ▲	⊕
XRCC1		-0.05	0.86	0.009 ▲	⊕
NCOA3		-0.05	0.64	0.009 ▲	⊕
VASP		0.04	-0.52	0.009 ▼	⊕
CHEK1		-0.03	0.49	0.010 ▲	⊕
RAB25		0.03	-0.23	0.011 ▼	⊕
BCL2L1		0.04	-0.53	0.012 ▼	⊕
IGF1R		-0.03	0.49	0.013 ▲	⊕
FN1		0.03	-0.50	0.014 ▼	⊕
RAD51		-0.03	0.40	0.015 ▲	⊕
PRKCA		0.02	-0.39	0.015 ▼	⊕
CHEK2		-0.03	0.35	0.016 ▲	⊕
PTK2		0.03	-0.44	0.017 ▼	⊕
TP53		-0.03	0.46	0.019 ▲	⊕
NOTCH3		-0.04	0.58	0.019 ▲	⊕
TP53BP1		-0.04	0.52	0.022 ▲	⊕
GSK3A/GSK3B		-0.03	0.41	0.026 ▲	⊕
BCL2L1		0.02	-0.37	0.026 ▼	⊕
CD49		0.03	-0.41	0.028 ▼	⊕
MAPK1		0.02	-0.44	0.034 ▼	⊕
GATA3		0.03	-0.38	0.039 ▼	⊕
GAB2		0.02	-0.38	0.043 ▼	⊕
ERCC1		-0.02	0.28	0.045 ▲	⊕
CDH1		0.03	-0.53	0.048 ▼	⊕

Antibody Type: phosphorylation   Search:

Target		Ave. Abundance		p-value	Plot
Protein	Residue	Unaltered	Altered		
WWTR1	pS89	-0.07	0.96	4.73e-5 ▲	⊕
FOXO3	pS318	0.03	-0.58	3.50e-4 ▼	⊕
ARAF	pS299	0.04	-0.58	3.75e-4 ▼	⊕
RB1	pS807	-0.05	0.71	0.004 ▲	⊕
SHC1	pY317	0.03	-0.46	0.004 ▼	⊕
CHEK2	pT68	-0.02	0.46	0.013 ▲	⊕
JUN	pS73	-0.04	0.53	0.016 ▲	⊕
CHEK1	pS345	-0.02	0.39	0.022 ▲	⊕
EIF4EBP1	pT170	-0.03	0.50	0.037 ▲	⊕
RPS6KA1	pT359	-0.03	0.40	0.043 ▲	⊕

Konovalov and Garcia-Bassets, **Figure S5**
