# Supplementary material for: Analysis of the levels of lysine-specific demethylase 1 (LSD1) mRNA in human ovarian tumors and the effects of chemical LSD1 inhibitors in ovarian cancer cell lines
Source: J Ovarian Res. 2013 Oct 29;6:75. doi: 10.1186/1757-2215-6-75 (PMC4176291; doi:10.1186/1757-2215-6-75)
Supplement: Additional file 8: Figure S6 — Analysis of cell viability upon treatment with chemical LSD1 inhibitors in a panel of ovarian cancer lines. (A) Chemical structures of LSD1 inhibitors CAS 927019-63-4 and CBB1007. (B-E) MTS/PMS viability assay in SKOV3, OVCAR-3, A2780, and A2780cis cells treated with different LSD1 inhibitors: pargyline (red), TCP (orange), RN-1 (light blue), and S2101 (dark blue). Measurements are shown as dots. Error bars represent s.e.m. Lines represent the estimated inhibitory curve response. The x-axis is log-10 scale. [file 1757-2215-6-75-S8.pdf]

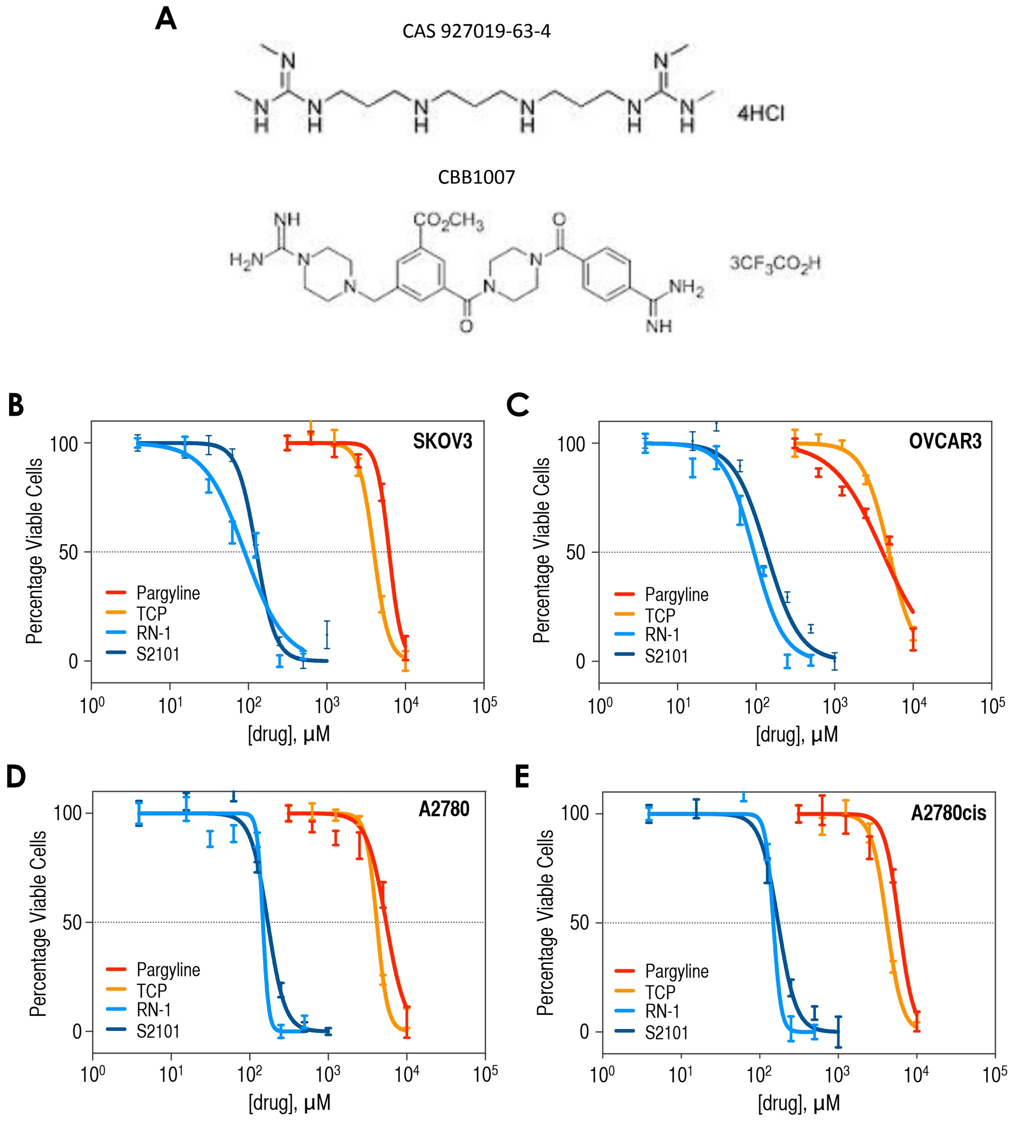

Konovalov and Garcia-Bassets, **Figure S6**
